# Deficits in Error-Monitoring by College Students with Schizotypal Traits: An Event-Related Potential Study

**DOI:** 10.1371/journal.pone.0122861

**Published:** 2015-03-31

**Authors:** Seo-Hee Kim, Kyoung-Mi Jang, Myung-Sun Kim

**Affiliations:** Department of Psychology, Sungshin Women’s University, Seoul, Republic of Korea; University of Manchester, UNITED KINGDOM

## Abstract

The present study used event-related potentials (ERPs) to investigate deficits in error-monitoring by college students with schizotypal traits. Scores on the Schizotypal Personality Questionnaire (SPQ) were used to categorize the participants into schizotypal-trait (*n* = 17) and normal control (*n* = 20) groups. The error-monitoring abilities of the participants were evaluated using the Simon task, which consists of congruent (locations of stimulus and response are the same) and incongruent (locations of stimulus and response are different) conditions. The schizotypal-trait group committed more errors on the Simon task and exhibited smaller error-related negativity (ERN) amplitudes than did the control group. Additionally, ERN amplitude measured at FCz was negatively correlated with the error rate on the Simon task in the schizotypal-trait group but not in the control group. The two groups did not differ in terms of correct-related potentials (CRN), error positivity (Pe) and correct-related positivity (Pc) amplitudes. The present results indicate that individuals with schizotypal traits have deficits in error-monitoring and that reduced ERN amplitudes may represent a biological marker of schizophrenia.

## Introduction

Error-monitoring is defined as the ability to monitor one’s own behavior, such as detecting errors and correcting or adjusting one’s behavior to achieve the intended purposes [1,2]. It has been consistently reported that patients with schizophrenia suffer from impaired error-monitoring [3–7]. Patients with schizophrenia exhibit significantly longer response times and commit more errors compared with normal controls [8,9]; these results may reflect that patients with schizophrenia cannot recognize erroneous behavior [10,11] or cannot adjust the erroneous behavior to achieve their intended purposes [4, 12].

Event-related potentials (ERPs), the electrical brain activity time-locked to external events or responses, are widely used to investigate cognitive functions, including error-monitoring, due to the high temporal resolution associated with this technique [13]. Several ERP studies have identified two electrophysiological indices of error-monitoring; one is designated as error-related negativity (ERN) and the other as error positivity (Pe) [1, 14–17]. ERN is a negative peak observed over fronto-central sites 50–150 ms after erroneous responses, and its functional significance is not yet fully understood. For example, it has been suggested that ERN reflects errors detected as a result of a comparison between the representations of the required and the executed responses [1], or indicates the magnitude of individuals’ responses to their own errors as well as error detection *per se* [16]. However, a fronto-central negative potential has also been observed after correct responses, which is referred to as the correct-related negativity (CRN) [8, 18]. Subsequent studies therefore have come to different conclusions regarding to the functional significance of ERN. For example, Vidal et al. [18] suggested that ERN may reflect the process of comparison that secondarily results in error detection, rather than an error detection process *per se*. Furthermore, Botvinick et al. [12] proposed that ERN is associated with the detection of response conflict. Although the functional significance of ERN remains controversial, a growing body of evidence suggests that ERN indexes general behavioral monitoring within the brain [1,4].

Previous studies have reported that patients with schizophrenia demonstrate reduced ERN and augmented CRN amplitudes relative to normal controls [7,8,19,20]. For example, Morris et al. [7] and Kim et al. [20] investigated error-monitoring in patients with schizophrenia using the Flanker task and the Stroop task, respectively, and found significantly reduced ERN and augmented CRN amplitudes in patients with schizophrenia than in normal controls. Furthermore, reduced ERN amplitudes have been observed in patients with first-episode schizophrenia, individuals at high-risk for schizophrenia [19], and healthy family members of patients [21] and persists even after schizophrenic symptoms are relieved [4]. Thus, reduced ERN amplitude is regarded as a trait-marker of schizophrenia.

Another ERP component explored within the context of error-monitoring is error positivity (Pe). Pe, which is seen after ERN, occurs in centro-parietal sites 150–400 ms after erroneous responses. Pe has been less studied than has ERN, and its functional significance remains poorly understood. Some investigators have suggested that Pe is the delayed P300 that reflects information updating. For example, Leuthold and Sommer [22] found a significant association between Pe and P300 and suggested that Pe indicates error recognition and updating of erroneous context. However, other investigators have suggested that Pe is not the delayed P300 but rather reflects the conscious recognition of errors and the motivation to correct the errors [23–26]. A positive potential, referred to as the correct-related positivity (Pc), has also been observed subsequent to the onset of correct responses [8] but its cognitive function is little known [4]. Previous studies have reported inconsistent findings with respect to Pe/Pc amplitudes in patients with schizophrenia: some studies found that patients with schizophrenia did not differ from normal controls in Pe/Pc amplitudes [4,8,20], whereas others reported reduced Pe amplitudes in chronic and first-episode patients with schizophrenia relative to normal controls [19].

The anterior cingulate cortex (ACC) is known as the generator of ERN and Pe [27–29]. Neuroimaging studies have identified the neuroanatomical basis for the error-monitoring deficits observed in patients with schizophrenia in terms of the structural and functional abnormalities of the ACC of these patients [30–32]. For example, Poli et al. [9] observed reduced activation of the ACC while patients with schizophrenia were committing errors and suggested that this indicates that these patients experience difficulties in detecting errors.

The tasks used for the measurement of error-monitoring should elicit a significant number of errors [8] and the errors should be elicited by slips due to the rapid responding required by the task but not by mistakes resulting from misunderstanding the task [2,33]. The Simon task is widely used for the measurement of error-monitoring because this task can elicit errors by slips through manipulating compatibility [34]. The Simon task, based on the association between the locations of stimulus and response, consists of congruent and incongruent conditions. The locations of stimulus and response are the same under the congruent condition, whereas the locations of stimulus and response are different under the incongruent condition [35]. Longer response times and more errors are observed under the incongruent than the congruent condition, and this phenomenon is called the Simon effect [34,35]. The Simon effect reflects the process of committing errors even though the subject knows the correct response due to the rapid responding required in this task [36]. Previous studies have reported that patients with schizophrenia exhibited longer response times and made more errors (i.e., a higher Simon effect [37]) or reduced ERN [38] compared with normal controls

Given that schizophrenia is highly heterogeneous and that several factors including antipsychotic drugs and length of illness or hospitalization can affect performance, investigation of the endophenotypes associated with schizotypal personality disorder (SPD) and with schizotypal traits in non-clinical individuals appears to be a promising approach to understanding schizophrenia [39]. Indeed, SPD and schizophrenia share common genetic [40], neuroimaging [41], and neuropsychological [42] abnormalities. Furthermore, difficulties in error-monitoring are present in individuals with psychometrically defined schizotypal traits [43,44].

Thus, the present study used ERPs and the Simon task to investigate the ability of college students with schizotypal traits to monitor errors. Based on previous findings, we hypothesized that individuals with schizotypal traits would exhibit longer response times, commit more errors on the Simon task, and demonstrate reduced ERN and Pe amplitudes compared with normal controls. To our knowledge, ours is the first study to use ERPs and the Simon task to investigate error-monitoring in non-clinical individuals with schizotypal traits.

## Materials and Methods

### Ethics Statement

The participants were paid for their participation, and provided written informed consent after receiving a complete description of the study. This study was approved by the Sungshin Women’s University Institutional Bioethics Review Board (sswuirb-2013-005).

### Participants

The present study included 37 college students who were recruited from a pool of 503 students and screened with the Korean version of the Schizotypal Personality Questionnaire [45,46]. The SPQ is a 74-item self-administered questionnaire with a “yes/no” response format. All items answered “yes” are scored as one; therefore, the total maximum score is 74. The factor analysis study of the SPQ showed that SPQ consists of three factors, such as cognitive-perceptual, interpersonal, and disorganized factors [47]. Students in the top 5% of total SPQ scores were included in the schizotypal-trait group (*n* = 17; eight males and nine females), whereas the control group (*n* = 20; ten males and ten females) was comprised of students with average scores (± 1 *SD*) on this instrument [46,48]. The Structured Clinical Interview for DSM-Ⅳ, Non-Patient version (SCID-NP, [49]) was administered to ensure that the participants did not have a history of psychiatric, medical or neurological disorders or drug/alcohol abuse. All participants were right-handed and none was taking medication at the time of testing.

### Simon Task

**Fig 1:**
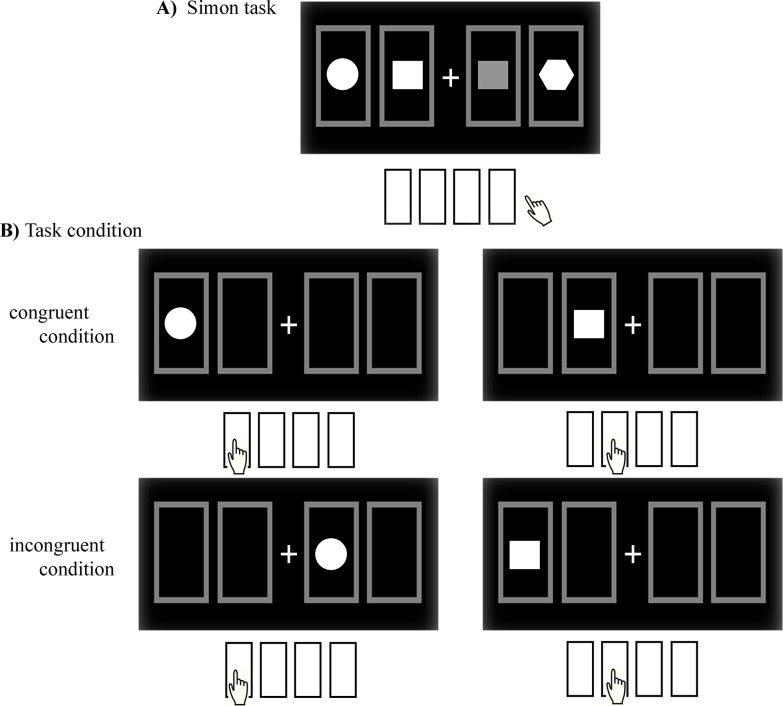
The Simon task. A) Four stimuli (a circle, a bright square, a dark square, and a pentagon) were presented, and a specific response location was designated for each stimulus. B) The Simon task consists of a congruent condition, under which the locations of stimulus and response are the same, and an incongruent condition, under which the locations of stimulus and response are different.

The Simon task was administered to measure error-monitoring. Four stimuli (i.e., a circle, a bright square, a dark square, and a pentagon) were presented, and a specific response location was designated for each stimulus (Fig 1A). The stimulus was randomly presented on either the left or right side of a fixation cross (+) that appeared at the center of the computer monitor, and participants were asked to respond by pressing the button associated with the stimulus as rapidly and accurately as possible. The Simon task consists of a congruent condition, under which the locations of the stimulus and the response-button designated for that stimulus are the same, and an incongruent condition, under which the locations of the stimulus and the button designated for that stimulus are different (Fig 1B). Thus, one congruent and three incongruent conditions could be presented, but this study presented both the congruent and incongruent conditions (360:360 trials) and the three incongruent conditions at the same rate (120:120:120 trials). A total of 780 trials were administered randomly in three blocks and the associations between the stimulus and its response position were changed across the blocks.

The stimulus was presented for 800 ms on the left or right side of the fixation cross that appeared at the center of a computer monitor; E-PRIME (Psychological Software Tools Inc., Sharpsburg, PA, USA) was used for these operations. The crosshair was displayed for 700 ms and the inter-stimulus interval was 1980 ms. Prior to the experimental session a block of 60 trials was administered to ensure that the instructions were understood.

### Electrophysiological Recording Procedure

Electroencephalographic activity (EEG) was recorded using a 64-channel HydroCel Geodesic Sensor Net connected to a 64-channel, high-input impedance amplifier (Net Amp 300: Electrical Geodesics, Eugene, OR, USA) in an electrically shielded and soundproofed experimental room. Each electrode was referenced to Cz, and individual electrodes were adjusted until impedances were less than 50 kΩ [50]. Eye movements and blinks were monitored with electrodes placed near the outer canthus and beneath the left eye.

During the experiment, EEG activity was recorded continuously using a 0.1–100 Hz analog bandpass and a sampling rate of 250 Hz. Following data collection, the EEG data were segmented into an epoch that started 100 ms before the onset of response and lasted until 500 ms after the onset of response. Epochs contaminated by artifacts, such as eye blinks and eye movements, were rejected prior to the averaging (the threshold for artifact rejection was ± peak-to-peak amplitude of 70 uV). All data associated with incorrect and correct trials were averaged separately with an average-reference transformation, and the ERPs were digitally low-pass filtered at 30 Hz. The mean number of trials included in the ERN/Pe analysis for the schizotypal-trait and control groups were 55.18 (*SD* = 10.31) and 44.35 (*SD* = 8.37), respectively. The mean number of trials for CRN/Pc were 434.53 (*SD* = 54.17) and 498.34 (*SD* = 48.34) for the schizotypal-trait and the control group, respectively. The two groups did not differ in terms of trials for both ERN/Pe averaging (*t*(35) = -1.04, ns) and CRN/Pc averaging (*t*(35) = 1.28, ns).

### Statistical Analysis

**Fig 2:**
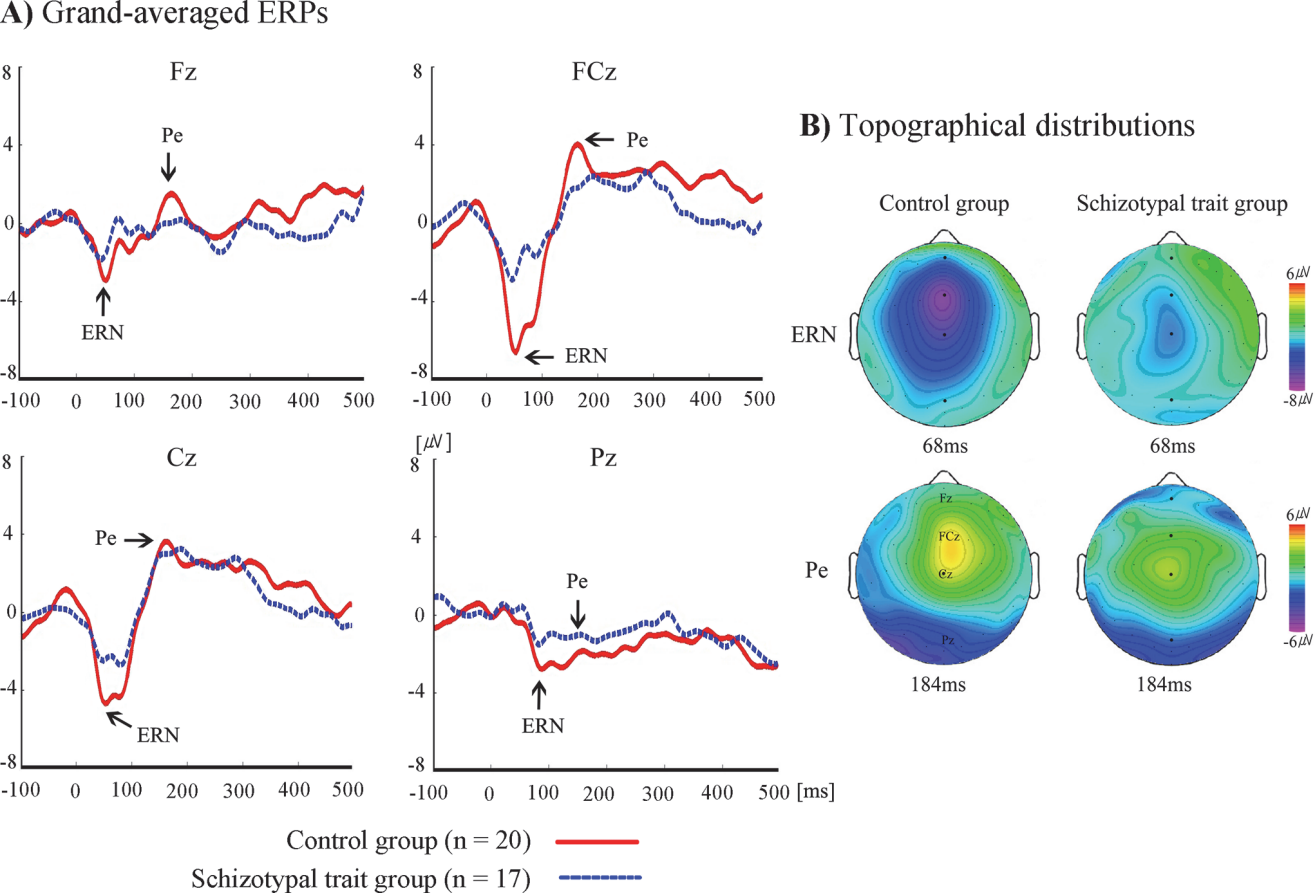
The grand-averaged ERPs and topographical distributions. A) The grand-averaged ERPs elicited by erroneous responses at Fz, FCz, Cz, and Pz for schizotypal-trait and control groups. B) Topographical distributions of ERN and Pe elicited by erroneous responses for schizotypal-trait and control groups.

**Fig 3:**
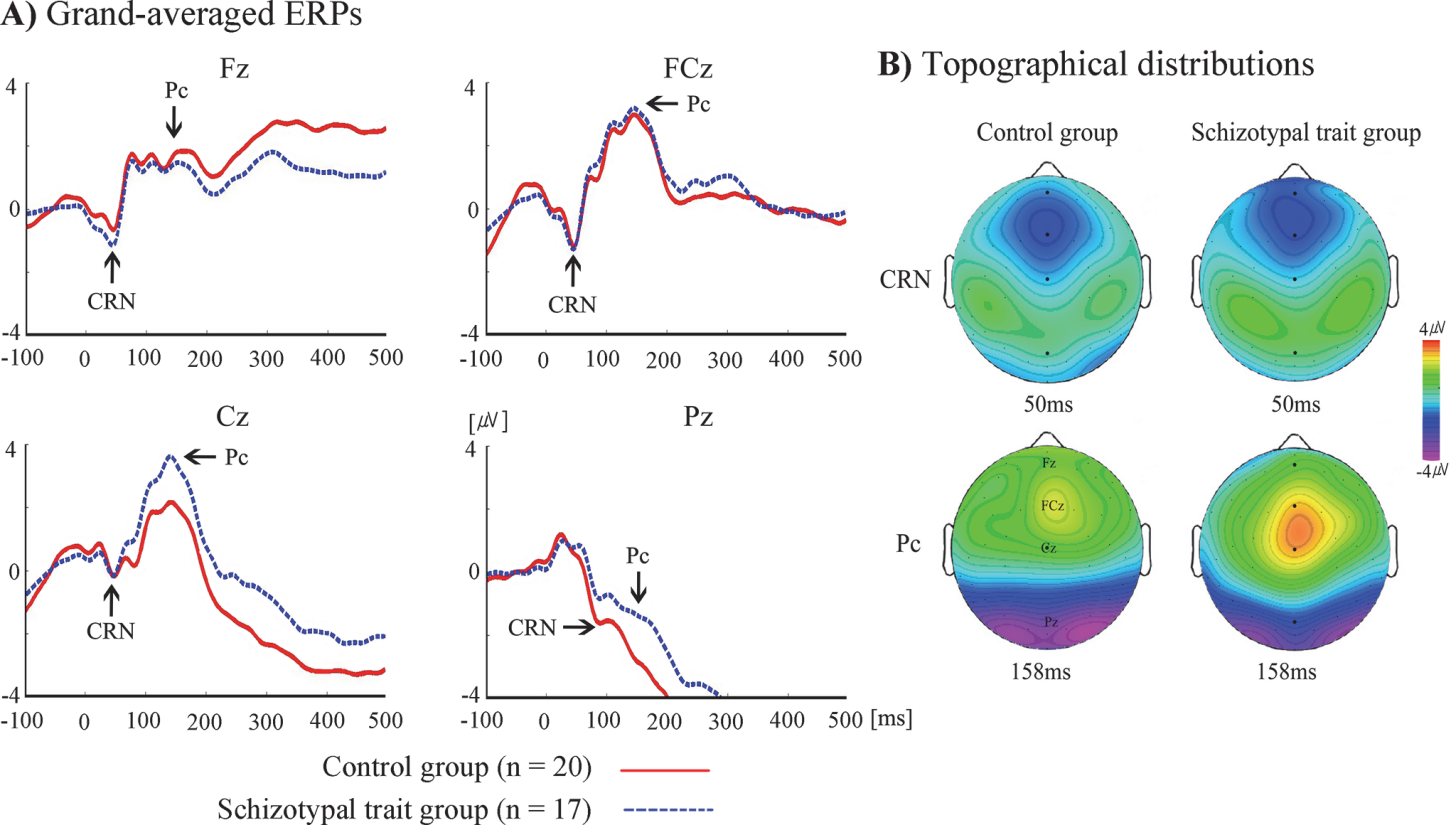
The grand-averaged ERPs and topographical distributions. A) The grand-averaged ERPs elicited by correct responses at Fz, FCz, Cz, and Pz for schizotypal-trait and control groups. B) Topographical distributions of CRN and Pc elicited by correct responses for schizotypal-trait and control groups.

The time windows of ERN/Pe and CRN/Pc were determined following a visual inspection of the grand-averaged (Fig 2A and Fig 3A) and individual ERP waveforms. ERN/CRN were defined as the most negative peaks observed 50–150 ms after the onset of incorrect and correct responses, respectively, and Pe/Pc were defined as the most positive peaks observed 150–300 ms after the onset of incorrect and correct responses, respectively. The amplitudes and latencies of ERN/Pe and CRN/Pc were analyzed with mixed-design repeated measures analysis of variance (ANOVA) using electrode site as a within-subject factor (F3, F4, Fz, FC3, FC4, FCz, C3, C4, Cz, P3, P4 and Pz) and group (schizotypal-trait and control groups) as a between-subjects factor. In addition to midline electrode sites (Fz, FCz, Cz, and Pz), left (F3, FC3,C3, and P3) and right (F4, FC4, C4, and P4) sites were included in the analysis since a laterality effect on the Simon task in patients with schizophrenia was observed [51]. Green-Geisser corrections for violations of sphericity were used when appropriate, and the correct *p* values are reported. The associations between ERN/Pe and CRN/Pc amplitudes and error rates on the Simon task were examined using Pearson product-moment correlations. The demographic characteristics and the behavioral performances (response time and number of errors) were analyzed by means of independent *t*-tests.

## Results

### Demographic Characteristics

The demographic characteristics and SPQ scores of the schizotypal-trait and control groups are presented in Table 1. The two groups did not differ in terms of mean age (*t*(35) = .71, ns) or years of education (*t*(35) = .95, ns). Additionally, the total IQ score of the two groups, measured by the Korean-Wechsler Adult Intelligence Scale [52], did not differ (*t*(35) = .93, ns). However, the SPQ scores of the two groups differed significantly, and the schizotypal-trait group obtained significantly higher total scores on the SPQ compared with the control group (*t*(35) = -18.69, *p* <. 001). In addition, the schizotypal-trait group exhibited significantly higher cognitive-perceptual (*t*(35) = -6.70, *p* <. 001), interpersonal (*t*(35) = -8.48, *p* <. 001) and disorganized (*t*(35) = -5.50, *p* <. 001) factors compared with the control group.

**Table 1:** Demographic characteristics of normal control and schizotypal-trait groups.

	Normal control (*n* = 20)	Schizotypal-trait (*n* = 17)
	Mean (SD)	Mean (SD)
Age (years)	21.40 (1.98)	21.00 (1.46)
Educational level (years)	14.55 (0.95)	14.82 (0.81)
IQ	111.80 (5.91)	110.18 (4.41)
SPQ (total)ᵃ	17.70 (1.66)	41.65 (5.45)
Cognitive-perceptual factor	8.85 (3.07)	16.88 (4.21)
Interpersonal factor	6.30 (3.74)	19.29 (5.53)
Disorganized factor	3.80 (2.12)	8.76 (3.33)

ᵃSPQ = Schizotypal Personality Questionnaire.

### Behavioral Performance on the Simon task

The mean response times of the schizotypal-trait and control groups were 571.90 ms (*SD* = 77.91) and 567.24 ms (*SD* = 60.06), respectively, and the two groups did not differ in terms of response time (*t*(35) = -.19, ns). The mean number of errors between the two groups differed significantly (*t*(35) = -2.10, *p* <. 05), and the schizotypal-trait group exhibited more errors (*M* = 92.94: range = 140–55) compared with the control group (*M* = 62.25: range = 98–41).

### Electrophysiological Measures

Fig 2A displays the grand-averaged ERPs elicited by incorrect responses at Fz, FCz, Cz and Pz. The ERN and Pe were observed for both schizotypal-trait and control groups, and the schizotypal-trait group showed reduced ERN and Pe amplitudes, particularly at the FCz site, compared with the control group. Fig 2B depicts the topographical distributions of electrical activity measured at all 64 sites at the time the maximum ERN and Pe amplitudes were observed. Fig 3A displays the grand-averaged ERPs elicited by correct responses at 4 midline electrode sites, and the CRN and Pc were also observed for both groups. Fig 3B depicts the topographical distributions of electrical activity at the time the maximum CRN and Pc amplitudes were observed.

Analysis of the ERN amplitudes revealed the main effects of group (*F*(1, 35) = 7.37, *p* <. 05, Ƞ_p_^2^ = .174) and electrode site (*F*(11, 385) = 16.02, *p* <. 05, Ƞ_p_^2^ = .314). The schizotypal-trait group exhibited a significantly smaller ERN amplitude (*M* = -2.18 uV, *SD* = 1.48) than did the control group (*M* = -3.51 uV, *SD* = 1.48). In terms of electrode site, the largest ERN amplitude was observed at FCz (*M* = -5.99 uV, *SD* = 3.52) and the smallest amplitude at P4 (*M* = -1.48 uV, *SD* = 2.04). There was no significant interaction effect of group by electrode site (*F*(11, 385) = 1.72, ns, Ƞ_p_^2^ = .079). With respect to ERN latency, only a main effect of electrode site was observed (*F*(11, 385) = 25.22, *p* <. 001, Ƞ_p_^2^ = .419). The shortest ERN latency was observed at FC4 (*M* = 59.64 ms, *SD* = 19.16), and the longest one was observed at P4 (*M* = 83.96 ms, *SD* = 13.46). No significant main effect of group (*F*(1, 35) = 1.77, ns, Ƞ_p_^2^ = .048) or interaction effect of group by electrode site (*F*(11, 385) = .21, ns, Ƞ_p_^2^ = .006) was observed. The mean ERN amplitudes and latencies for the schizotypal-trait and control groups are presented in Table 2.

**Table 2:** Mean ERN amplitudes and latencies in normal control and schizotypal-trait groups.

site	Normal control (*n* = 20)		Schizotypal-trait (*n* = 17)	
	Amplitude (*μ*v)	Latency (ms)	Amplitude (*μ*v)	Latency (ms)
F3	-3.26 (3.75)	64.50 (19.73)	-1.70 (2.64)	58.82 (18.32)
Fz	-3.76 (3.41)	65.30 (18.89)	-2.48 (1.91)	57.88 (16.85)
F4	-2.23 (2.89)	61.40 (19.43)	-1.90 (1.99)	59.05 (19.06)
FC3	-3.93 (2.55)	62.90 (18.55)	-1.98 (1.10)	56.82 (17.09)
FCz	-7.86 (4.21)	63.95 (19.07)	-4.12 (2.41)	61.29 (20.41)
FC4	-2.91 (2.33)	63.45 (21.38)	-1.68 (1.75)	55.82 (15.96)
C3	-3.08 (2.39)	65.75 (17.09)	-1.32 (1.48)	59.00 (18.59)
Cz	-5.92 (2.50)	64.10 (17.55)	-3.25 (2.41)	56.82 (16.61)
C4	-1.61 (1.90)	65.35 (19.43)	-1.72 (2.19)	58.53 (17.19)
P3	-2.86 (2.60)	85.70 (13.15)	-2.25 (1.55)	80.00 (13.70)
Pz	-2.91 (1.83)	82.90 (17.45)	-2.60 (1.48)	77.88 (14.73)
P4	-1.79 (1.88)	87.35 (13.16)	-1.17 (1.40)	80.58 (13.70)

() standard deviation.

A main effect of electrode site was observed (*F*(11, 385) = 7.71, *p* <. 001, Ƞ_p_^2^ = .181) with regard to CRN amplitude. The largest CRN amplitude was observed at FCz (*M* = -1.35 uV, *SD* = 2.48) and the smallest one at C4 (*M* = .46 uV, *SD* = 1.49). No significant effect of group (*F*(1, 35) = .01, ns, Ƞ_p_^2^ = .000) or interaction effect of group by electrode site (*F*(11, 385) = .78, ns, Ƞ_p_^2^ = .022) was observed. With respect of CRN latency, only a main effect of electrode site was observed (*F*(11, 385) = 128.65, *p* <. 001, Ƞ_p_^2^ = .786). The shortest CRN latency was observed at F4 (*M* = 44.54 ms, *SD* = 3.80), and the longest one was observed at P3 (*M* = 84.46 ms, *SD* = 8.97). No significant main effect of group (*F*(1, 35) = .66, ns, Ƞ_p_^2^ = .018) or interaction effect of group by electrode site (*F*(11, 385) = 2.35, ns, Ƞ_p_^2^ = .063) was observed. The mean CRN amplitudes and latencies for the schizotypal-trait and control groups are presented in Table 3.

**Table 3:** Mean CRN amplitudes and latencies in normal control and schizotypal-trait groups.

site	Normal control (*n* = 20)		Schizotypal-trait (*n* = 17)	
	Amplitude (*μ*v)	Latency (ms)	Amplitude (*μ*v)	Latency (ms)
F3	-0.38 (1.24)	49.45 (8.35)	-0.79 (2.14)	44.76 (3.68)
Fz	-0.75 (1.66)	46.05 (1.85)	-1.28 (2.34)	45.47 (2.35)
F4	-0.24 (1.37)	44.55 (4.05)	-0.85 (1.88)	44.53 (3.45)
FC3	-0.17 (1.53)	46.55 (2.35)	-0.37 (1.23)	45.35 (2.26)
FCz	-1.34 (2.67)	46.20 (2.33)	-1.37 (2.23)	45.88 (1.96)
FC4	-0.18 (1.47)	45.75 (3.29)	-0.09 (1.46)	46.29 (3.29)
C3	0.29 (1.62)	61.55 (17.63)	0.18 (0.88)	53.71 (15.51)
Cz	-0.54 (2.88)	56.25 (14.63)	-0.46 (1.69)	50.88 (10.96)
C4	0.39 (1.68)	57.60 (14.29)	0.54 (1.22)	66. 82 (15.17)
P3	-1.21 (1.83)	85.45 (6.53)	-1.02 (0.99)	83.47 (11.15)
Pz	-1.65 (2.79)	84.70 (5.53)	-0.90 (1.02)	81.94 (10.51)
P4	-1.12 (1.76)	80.30 (10.88)	-0.89 (1.00)	83.06 (6.50)

() standard deviation.

A main effect of electrode site was observed (*F*(11, 385) = 17.22, *p* <. 001, Ƞ_p_^2^ = .330) with regard to Pe amplitude. The largest Pe amplitude was observed at Cz (*M* = 4.74 uV, *SD* = 3.58), and the smallest amplitude at Pz (*M* = -0.31 uV, *SD* = 2.41). However, no significant main effect of group (*F*(1, 35) = 1.13, ns, Ƞ_p_^2^ = .031) or interaction effect of group by electrode site (*F*(11, 385) = 1.67, ns, Ƞ_p_^2^ = .046) was observed. In terms of Pe latency, a main effect of electrode site was observed (*F*(11, 385) = 5.51, *p* <. 001, Ƞ_p_^2^ = .137), with the shortest latency at Cz (*M* = 175.43 ms, *SD* = 24.76) and the longest at Pz (*M* = 189.95 ms, *SD* = 24.74). No significant main effect of group (*F*(1, 35) = 1.75, ns, Ƞ_p_^2^ = .047) or interaction effect of group by electrode site (*F*(11, 385) = .39, ns, Ƞ_p_^2^ = .011) was observed in terms of Pe latency. The mean Pe amplitudes and latencies for the schizotypal-trait and control groups are presented in Table 4.

**Table 4:** Mean Pe amplitudes and latencies in normal control and schizotypal-trait groups.

site	Normal control (*n* = 20)		Schizotypal-trait (*n* = 17)	
	Amplitude (*μ*v)	Latency (ms)	Amplitude (*μ*v)	Latency (ms)
F3	1.37 (3.20)	176.50 (27.29)	1.77 (2.50)	182.47 (24.08)
Fz	2.54 (3.48)	169.40 (13.38)	1.27 (3.13)	181.24 (23.77)
F4	2.74 (2.61)	176.20 (24.54)	0.94 (2.32)	181.24 (15.83)
FC3	2.63 (2.09)	172.95 (24.66)	2.19 (1.33)	182.35 (24.21)
FCz	5.14 (4.29)	173.45 (21.16)	3.41 (3.06)	180.88 (25.84)
FC4	3.75 (1.79)	173.90 (22.99)	2.64 (2.10)	181.71 (20.56)
C3	1.83 (1.85)	172.75 (25.91)	2.14 (1.54)	181.53 (26.27)
Cz	4.52 (3.96)	170.10 (17.87)	4.95 (3.02)	180.76 (30.86)
C4	2.85 (2.01)	174.40 (26.13)	1.90 (1.67)	182.18 (20.65)
P3	0.27 (1.60)	180.45 (20.98)	0.32 (2.57)	194.41 (26.47)
Pz	-0.84 (2.56)	187.60 (20.92)	0.21 (2.19)	192.29 (28.45)
P4	1.00 (2.01)	183.30 (24.53)	-0.11 (1.58)	193.47 (22.58)

() standard deviation.

Analysis of the Pc amplitude revealed a main effect of electrode site (*F*(11, 385) = 31.23, *p* <. 001, Ƞ_p_^2^ = .472). The largest Pc amplitude was observed at FCz (*M* = 3.41 uV, *SD* = 3.30) and the smallest one at Pz (*M* = -1.78 uV, *SD* = 2.90). However, no significant effect of group (*F*(1, 35) = .11, ns, Ƞ_p_^2^ = .003) or interaction effect of group by electrode site (*F*(11, 385) = 1.03, ns, Ƞ_p_^2^ = .029). With respect of Pc latency, a main effect of electrode site was observed (*F*(11, 385) = 4.51, *p* <. 01, Ƞ_p_^2^ = .114), with the shortest latency at FCz (*M* = 151.96 ms, *SD* = 11.06) and the longest at C4 (*M* = 163.20 ms, *SD* = 10.37). No significant main effect of group (*F*(1, 35) = 3.28, ns, Ƞ_p_^2^ = .086) or interaction effect of group by electrode site (*F*(11, 385) = 1.05, ns, Ƞ_p_^2^ = .029) was observed in terms of Pe latency. The mean Pc amplitudes and latencies for the schizotypal-trait and control groups are presented in Table 5.

**Table 5:** Mean Pc amplitudes and latencies in normal control and schizotypal-trait groups.

site	Normal control (*n* = 20)		Schizotypal-trait (*n* = 17)	
	Amplitude (*μ*v)	Latency (ms)	Amplitude (*μ*v)	Latency (ms)
F3	1.60 (2.01)	164.20 (13.59)	1.04 (2.76)	157.18 (10.16)
Fz	2.26 (1.95)	161.00 (14.99)	1.70 (2.86)	157.65 (10.31)
F4	2.09 (1.48)	165.95 (13.66)	1.59 (1.88)	157.18 (11.54)
FC3	1.93 (2.13)	157.85 (10.39)	1.87 (2.36)	153.53 (11.93)
FCz	3.36 (3.36)	153.15 (12.16)	3.46 (3.20)	150.76 (9.51)
FC4	2.26 (1.59)	163.45 (10.99)	2.49 (2.16)	155.12 (10.42)
C3	1.58 (2.10)	163.25 (10.08)	1.58 (2.34)	155.00 (12.61)
Cz	2.32 (4.17)	154.05 (11.73)	3.44 (4.22)	151.00 (9.86)
C4	1.62 (2.02)	165.35 (10.51)	1.64 (1.79)	161.06 (10.11)
P3	-1.50 (2.05)	158.45 (11.09)	-0.79 (1.71)	157.00 (12.91)
Pz	-2.46 (3.47)	156.00 (14.76)	-1.11 (1.98)	156.59 (12.04)
P4	-1.66 (2.00)	160.75 (13.52)	-1.23 (1.41)	162.00 (12.15)

() standard deviation.

### Correlations between the Amplitudes of ERN/Pe, CRN/Pc and Error Rates on the Simon Task

The ERN amplitude measured at FCz was negatively correlated with error rate on the Simon task in the schizotypal-trait group (*r* = -.59, *p* <. 05). However, no significant correlations between ERN/Pe, CRN/Pc amplitudes and error rate on the Simon task were observed in the normal control group.

## Discussion

The present study used ERPs and the Simon task to investigate whether college students with schizotypal traits exhibited deficits in error-monitoring. Compared with the control group, the schizotypal-trait group committed significantly more errors on the Simon task and exhibited smaller ERN amplitudes but comparable Pe amplitudes. The present findings are consistent with previous studies investigating the error-monitoring abilities of patients with schizophrenia, which have reported more errors on the Simon task [37] and reduced ERN amplitudes but comparable Pe amplitudes in patients with schizophrenia [8, 20, 38]. Additionally, Simmonite et al. [21] found reduced ERN amplitudes but comparable Pe amplitudes not only in patients with schizophrenia but also in healthy family members of patients relative to normal controls.

As ERN is associated with error detection or an internal comparison process [1], the reduced ERN amplitudes in individuals with schizotypal traits may indicate that these individuals have difficulties in the monitoring of internal behavior. The anterior cingulate cortex (ACC), which is involved in behavioral monitoring or inhibition, is known as the generator of the ERN [28,30,53–55]. For example, Ullsperger and von Cramon [56] found that erroneous responses elicited greater ACC activation than did correct responses, and Herrmann et al. [53] employed source localization analysis to observe that ERN and Pe are generated in the ACC. In addition, structural and functional abnormalities in the ACC are frequently observed in patients with schizophrenia [6,30,57], SPD [58], and non-clinical individuals at high-risk for developing schizophrenia [59]. These neuroimaging studies further support the idea that reduced ERN amplitudes indicate dysfunctional error-monitoring. In addition, the ERN amplitudes measured at FCz were negatively correlated with error rates in the Simon task in the schizotypal-trait group but not the control group. In other words, more errors committed on the Simon task means that ERN amplitude is reduced in individuals with schizotypal traits. And these findings are consistent with those of previous studies, which found that the ERN amplitude is smaller when paticipants make more errors [60, 16]. The schizotypal-trait and control groups did not differ in terms of response time on the Simon task. Previous studies have reported that patients with schizophrenia exhibited significantly longer response time on the Simon task relative to controls [37, 51]. These inconsistent findings may be related to methodological differences such as the participants employed in this study.

Previous studies have reported that patients with schizophrenia demonstrate augmented CRN amplitudes in addition to reduced ERN amplitudes relative to normal controls [7,8,19,20]. However, in the present study the schizotypal-trait and control groups did not differ in CRN amplitudes and these inconsistent findings seem to be related to differences in the characteristics of the participants in the studies. For sample, Perez et al. [19] observed augmented CRN amplitude only in chronic patients but not in clinical high risk patients, and the longer illness duration was associated with larger CRN amplitude. The authors suggested that these results indicate that CRN abnormality appears to worsen progressively over the illness course.

Taken together, these findings indicate that the reduced ERN amplitudes observed in individuals with schizotypal traits are likely related to structural/functions abnormalities in the ACC, and probably reflect error-monitoring deficits in these individuals. Furthermore, the reduced ERN amplitude but maintained CRN amplitude observed in the schizotypal-trait group indicate that individuals with schizotypal traits are indifferent or to have a lesser degree of responsiveness to their own errors compared to normal controls and reduced ERN amplitude could serve as a trait marker of schizophrenia.

The schizotypal-trait and control groups did not differ in terms of Pe amplitude, and there was no significant association between Pe amplitudes and behavioral performance in the Simon task in both groups. Previous studies with patients with schizophrenia reported relatively inconsistent findings in terms of Pe amplitudes; some studies found comparable Pe amplitudes between patients with schizophrenia and normal controls [8,20,38], whereas others found reduced Pe amplitudes in patients with schizophrenia compared with controls [19]. It has been suggested that Pe reflects conscious error processing or a conscious awareness of erroneous responses, because Pe is more pronounced for perceived errors than for unperceived errors [17]. The similar Pe amplitudes observed in the schizotypal-trait and control groups in the present study may suggest that individuals with schizotypal traits perceived their errors in the same way as did the controls. Because the Simon task employed in the present study has the advantage of eliciting errors by slips rather than by misunderstanding the task, it seems to allow for the recognition and perception of erroneous responses by individuals with schizotypal traits. Because the functional significance of Pe is not fully understood and previous studies have yielded inconsistent findings, further study is needed to better understand the role of Pe in the process of error-monitoring.

The present study has several limitations that should be overcome in future studies. First, the inclusion of only a small number of participants limits the generalizability of the findings. Second, findings of structural/functional abnormalities in the ACC of individuals at high-risk for schizophrenia suggest that future studies should use both neuroimaging techniques and ERPs to enhance understanding of the neurophysiological mechanisms underlying error-monitoring deficits experienced by those with schizophrenia or schizo-spectrum disorders.

In conclusion, college students with schizotypal traits committed significantly more errors on the Simon task and exhibited smaller ERN amplitudes relative to controls. Moreover, the ERN amplitude measured at FCz was negatively correlated with the error rate on the Simon task in the schizotypal-trait group but not in the control group. These findings indicate that individuals with schizotypal traits have difficulties in error-monitoring and that reduced ERN amplitudes may serve as a biological marker or risk factor for schizophrenia.
